# The Institutional Worker

**Published:** 1913-08-09

**Authors:** 


THE
### INSTITUTIONAL WORKER
**Being a Special Supplement to "The Hospital"**

### OUR BUREAU OF INFORMATION.

### Rules for Correspondents.

1. Every letter must be accompanied by the coupon to be cut from the back cover (inside page) of THE HOSPITAL, current issue, and must contain the name and address of the correspondent with pseudonym for publication if desired. Replies by post cannot be given, save under exceptional circumstances at the Editor's discretion.

2. Letters from Approved Homes in reply to special needs published in the Bureau should state terms and full particulars, and be sent prepaid under cover to the Editor of the Bureau with name written across coupon for identification.

3. Proprietors of Homes which have not yet been entered on the List of Approved Homes, but have spare accommodation likely to suit special needs, are invited to write for an application form for registration. The fee for registration, which includes two announcements of the Home in the Bureau and other privileges, is 10s.

4. All communications to be addressed to the Editor of THE HOSPITAL, 28 Southampton Street, Strand, London, W.C., and marked "Bureau of Information."

### INSTITUTIONAL FACTS AND FIGURES.

### THE METHOD OF RECORDING ADMISSIONS.

LAST week we dealt with this subject by publishing one of the answers received to Question 1 for July. The Institution whose method was described is one requiring subscribers' letters. This week we publish a second selected answer describing the procedure and books in use at a special hospital where no "letters" are required. A comparison of the two methods will prove very instructive. The question was: State the character of your institution, and describe your system of recording the admission, treatment, and discharge of patients admitted to the wards. Give descriptions of the various forms in use for the purpose, the method of dealing with them in the process of registration, and the method of filing history and treatment sheets. Add your further remarks.

### Second Selected Answer.

A SPECIAL HOSPITAL WITHOUT "LETTERS."

THE institution I serve confines itself to the treatment of a particular disease, and is one of those that is therefore generally known as a "special" hospital. Treatment is gratuitous and no "letters" are required. The necessary qualifications are that the patient is suffering from the disease treated, and that lack of means prevents the applicant obtaining adequate relief elsewhere.

Chronic cases—*i.e.* those which do not admit of cure, but for which palliative treatment can be given—are admitted, and there are a certain number of beds allotted to such patients who may remain for life.

ADMISSION.

Admission to the wards is obtained by personal attendance at the out-patient department and a recommendation from the medical officer in charge, or, in the event of circumstances preventing such attendance, by means of a special medical certificate on a prescribed form. This certificate is submitted to the member of the staff attending out-patients on the day of its receipt, when, if he considers the case a suitable one, it is entered in the "application register." All cases recommended for admission are entered in this register, and admission orders are sent out from it by the house surgeons, as far as possible in the order of application, due regard being, of course, paid to urgent cases. When the case has been entered through the certificate of an outside medical man, notice is sent to the patient stating approximately when an order may be expected; ineligible cases are so informed.

PARTICULARS REQUIRED FROM APPLICANT.

All applicants at their first attendance at the hospital are required to give the following particulars, which are entered in the "inquiry officer's report book" and signed as correct by the applicant: Name, address, occupation, income, family, wages earned by family. In cases of married women or children the occupations and incomes of the husbands or parents respectively are entered. In the event of doubt as to eligibility from a financial point of view, the applicant is interviewed by the secretary, and in any further uncertainty the position is discussed by him with the medical officer in charge of out-patients for the day.

FORMS IN USE.

Having ascertained that the applicant is eligible from the point of means, the out-patient clerk hands the patient a case cover containing a "history sheet," on which is recorded name, address, occupation, age, date of attendance; also a card with patient's name, case-cover number, name of medical officer in charge, and the day of the week for future attendance. This card the patient is instructed to bring at any subsequent attendance. It should be mentioned that across the card is printed the request "If used for begging, please retain and enclose it to the secretary at the hospital." Particulars of the disease and medicine prescribed are entered on the history sheet by the medical officer, and the case sheet with its cover is left at the dispensary. The cover cases and cards are of a different colour for each of the medical staff attending to out-patients, and each cover is stamped with a number corresponding with that of the attendance card.

A card index is also kept with the patients' names filed alphabetically on cards giving the attendance-card number, register number, address, and divided into spaces for the dates of future attendances.

Each medical officer has a different coloured card for use in the card index cabinet.

A "daily register of out-patients" is kept, in which is recorded the names of patients attending each day, with the number of weeks' medicine and dressings given. New patients are numbered consecutively from the commencement of each year. It should be noted that this register number has no connection with that of the case-cover number, the latter being solely for the purpose of reference, while the latter is for ascertaining the number of patients for statistical purposes. A summary is entered each day of the number of new and old male and female cases separately, and the attendances made by each, these figures being subsequently entered in the "statistics book."

The "application book" contains the date of each entry for admission, name, address, and disease of patient, particulars as to which ward to be admitted, and medical officer under whose care to be placed.

Each patient on attending for admission is required to sign the particulars as stated in the "inquiry officer's report book," unless an attendance has been made as an out-patient, in which case this has already been done.

In addition the name and address of the nearest relative and the religion of the patient are obtained and entered on the admission form, which is filed. The object of ascertaining the religion of the patient is solely for the purpose of acquainting the ministers of the various religions who visit the hospital. A card authorising the sister of the ward to admit the patient is then signed on behalf of the secretary; this has to be returned to the office with the admission signed thereon by the sister.

"The in-patients' register" contains the following particulars: patient's register number—i.e., numerical order of admission commencing from January 1 in each year—date of admission, name, address, age, ward, disease, operation, by whom performed, result of treatment, date of discharge, number of days in hospital.

The "in-patients' history sheet" contains diagnosis of case, progress and treatment of disease, and nature of operation, with spaces for photo. album number, *x*-ray book number, path. report number, museum catalogue number, and also a small round space for register number.

These history sheets are issued by the registrar, who inserts the register number. On the discharge of a patient the history sheets are brought to the office, where particulars are entered in the in-patients' register and the folio number inserted below the register number on the history sheet, viz. :

Register No. 196
Folio No. 45

The absence of the number below would indicate that the history sheet had not passed through the office. The in-patients' register is, of course, indexed alphabetically. The history sheets are filed numerically in a vertical cabinet and a double card index kept of the names and numbers. Every year the sheets are bound into separate volumes for each member of the staff and a separate index classifying the patients by name and by disease is also bound.

Every patient attends the office before leaving the hospital, is asked whether satisfied with the treatment given, and requested to sign accordingly in the discharge book. Any complaint is reported to the committee and carefully investigated.

Daily "bed lists" are sent to the out-patient department, showing the number of beds occupied in each ward by the patients of each member of the staff, number of beds each has vacant, and indicating which beds are reserved for patients for whom admission orders have been sent. A daily list showing the names of the patients in each ward is exhibited in the hall.

The entries in the discharge book and the counterfoils of the death certificate books are numbered. By this means the number of patients in the hospital can be proved correct according to the books by the following method. Take the number of patients in the hospital at the beginning of the year, add the last number of the admission register, deduct the last discharge-book number and the last counterfoil death-certificate number.

REMARKS.

This hospital has never issued subscribers' "letters," and therefore every case is considered on its own merits. Applicants are thus saved the anxiety and trouble of obtaining recommendations, and subscribers are relieved of harassing appeals from applicants the eligibility of whom they have in the majority of cases no means of judging.

INQUIRY OFFICER'S REPORT BOOK.

The fact that patients are required to sign the information they give prevents misunderstanding and serves as a check on attempted abuse of the charity, for it is found that patients are careful of the particulars they give when they are aware that they will be called upon to sign their statements.

CLASSIFYING BY MEANS OF COLOURS.

The use of different colours for history-sheet covers, attendance and index cards is of great assistance in preventing mistakes in filing and for rapid identification purposes. For instance, a patient mistaking the day for attendance is at once detected by the official as soon as the card is produced.—"REPOOHMA."

### QUESTIONS for AUGUST.

AUGUST is a holiday-making time, and therefore the questions we are publishing for this month are of such a nature as to be easy to answer without recourse to reference books. Question 2 touches on a branch of philanthropy which is coming greatly to the fore, and practical workers in this field are invited to give their experience which cannot fail to be of considerable help to the many beginners whom this work is attracting. Question 1 should appeal to the secretariat of any institution, and their views, with the reasons for such views, will be welcome.

**Question 1.**

**Is it or is it not an advantage for a hospital to possess an addressograph? Give reasons, and if an advantage give particulars of cost.**

**Question 2.**

**Give a week's menu for dinners for mothers in connection with Schools for Mothers, with the daily cost for 20 people, bearing in mind the need for nourishing food and for strict economy.**

RULES.

The following rules must be observed:—

1. Contributions must be written on one side of the paper. They must bear the name and address of the sender and be accompanied by coupon to be cut from the back cover (inside page) of the current issue of THE HOSPITAL. A pseudonym must be chosen if the name is not to be published.
2. They must be addressed to the Editor of THE HOSPITAL, 28 & 29 Southampton Street, Strand, London, W.C., so as to reach him by the last day of the month for which the questions are set, and must be marked in the left-hand corner "Facts and Figures."

**A minimum payment of Five Shillings will be made for each published answer.**

### Analysing and Checking Accounts for Payment.

WE have received several interesting answers to our July question under this head, some of which we hope to publish next week.

**The Editor is prepared to make known without charge the needs of any who may be sick or in difficulties, and to guide them in making choice of Homes for treatment or convalescence. Reduced terms can often be arranged with Homes on the Approved List for those unable to pay full fees. No Home is included in the Approved List without medical and other testimony to its good management. Queries are specially invited from those who are engaged in any kind of philanthropical work. A new edition of the leaflet describing the purposes of the Bureau of Information is now ready and will be sent free of charge on application. For Rules for Correspondents see page 1.**

### THE EDITOR'S LETTER-BOX.

*EMPLOYMENT AND TRAINING.*

### Free Massage Training.

WE should certainly advise you to enter for a general training before taking up massage and electricity, and by doing so, you will find it possible at certain institutions to obtain this special training, or a part of it, free, as you desire. At the Northampton General Hospital, probationers are prepared for the examination of the Incorporated Society of Trained Masseuses, and at the Portsmouth Parish Infirmary, nurses remaining for a fourth year receive massage as well as other special training. There are other institutions, particulars of which you will find in the book "How to Become a Nurse," published by The Scientific Press, Ltd., 28-29 Southampton Street, Strand, W.C., price 2s. 3d. post free.—H. R.

*ACKNOWLEDGMENTS.*

J. H. V.—We are forwarding your soap-saver to the address named, in accordance with your request.

S. J. W.—Your application for registration on our List of Approved Homes is receiving our attention.

A. M. B.—We are sending you copies of THE HOSPITAL containing Lists of our Approved Homes, from which we trust that you will be able to select a suitable and less expensive Home for the case of neurasthenia.

C. T. B.—We are making inquiries on behalf of the little girl for whom you desire to obtain free sanatorium treatment; meanwhile we would suggest your applying to the Secretary, Manchester Hospital for Consumpton.

*THE SICK AND IN NEED.*

### Home Offered to Phthisical Nurse.

AN opportunity occurs at a Home in Surrey for phthisical children (early cases) for a nurse, who herself may be phthisical, to find a home and employment. A trained or partly trained nurse, who has been phthisical but is quite fit for ordinary work and who would be glad to work under open-air conditions and among children would be suitable. Board, lodging, laundry, and a salary would be given to such a nurse. In the alternative a nurse would be received who is not fit for ordinary work but yet could manage, say, five or six hours daily; she would receive board, lodging, laundry, medical attendance, and a certain amount of care and treatment, but no salary. There are two probationers, both slightly phthisical, whose work the nurse would be required to supervise, and also to do the usual work among the children; but as only early cases are taken there is no heavy nursing. This opportunity may appeal to a nurse requiring an open-air life who on leaving a sanatorium may find it difficult to get a post.—Replies to "C. P. M.," 168R.

### Editor's Notices.

**Contributions:**

Contributions should be written, or preferably typed, on one side of the paper only, and all articles sent in are accepted upon the distinct understanding that they are forwarded to The Hospital only.

The Editor cannot undertake to return MSS. not used, but when a stamped directed envelope is enclosed the MSS. may be returned if a special request is made.

Accepted articles and paragraphs of news will be paid for after publication at the scale rate.

**Address:**

To prevent delay all contributions and letters on editorial business must be addressed exclusively to the Editor, "The Hospital," 29 Southampton Street, Strand, London, W.C. It is important that this regulation shall be strictly observed.

**Correspondence:**

Correspondence on all subjects is invited. The name and address of correspondents must be given as a guarantee of good faith, but not necessarily for publication.

**Special Articles:**

Special articles are invited, and questions, inquiries, and paragraphs upon all matters relating to the work, administration and management of general, special, mental, fever, cottage and convalescent hospitals, sanatoria, homes, institutions, societies and organisations for the treatment or care of the sick, injured and dependants of all classes. Every matter affecting the interests and welfare of the staff of all grades working in these institutions will receive special consideration.

**Administrative Medicine, Research and Items of News:**

Special terms will be paid for approved articles on subjects in this wide field by experts who have made some section of it their special study and interest. We wish to give prominence to every movement tending to advance accuracy of diagnosis, efficiency in treatment, and the development of modern methods to eradicate disease and promote the welfare of the suffering.

**A Bureau of Information:**

We invite inquiries and applications for information and help in providing for that numerous class of sufferers who require special care or house-room which they cannot obtain in their own homes or secure for themselves. We cannot, however, prescribe, or recommend practitioners.

**Books for Review:**

Publishers are particularly requested to send advance proofs of any new books of importance whenever possible, as the Editor has made arrangements to publish immediate reviews on a new plan.

**Photographs, Plans, Blocks, and Illustrations:**

It is requested that wherever possible MSS. may be accompanied by illustrations in any of the above forms. The name of the sender and of the article to which it belongs should be written on the back of each photograph, drawing, or block for purposes of identification.

**Local Papers:**

Newspapers containing reports or news paragraphs should be marked and addressed to the Sub-Editor.

**The Coupon System:**

A coupon will be found at the bottom of the third inside page of the cover each week. This coupon must be attached to every question or inquiry to which an answer is desired in The Hospital.

### A Help to Appointments.

As a means of announcing vacancies in every department of Institutional work, the success of *The Institutional Worker* is evidenced by the ever-increasing number of advertisements that appear in this section of The Hospital. No more adequate proof of the value of *The Institutional Worker* can be desired than the fact that Authorities of the more important Institutions throughout the United Kingdom employ its columns again and again whenever they have a vacancy to fill upon their staffs. Moreover, it provides exceptional facilities for every class of announcement affecting Institutional Life and Work, and as a medium for securing appointments in any section of the Institutional, Health, and Sanitary Services it is a distinct help to the worker, since no other journal possesses such a comprehensive circulation in these directions as The Hospital. If you are seeking a post an announcement of twenty-one words, costing the small sum of 1s., will save you many hours of anxious thought, and certainly bring your requirements immediately to the notice of those most likely to need your services.

The form below may be used for Appointments Wanted advertisements, and when filled up should be posted to *The Manager*, The Hospital,
28 and 29 Southampton Street,
Strand, W.C.

Appointments Wanted, 21 words ... 1s. 0d.

| | | |
|---|---|---|
| | | |
| | | |
| | | |
| | | |
| | | |
| | | |
| | | |

### Manager's Notices.

**Letters relating to the publication, sale, and advertising departments of "The Hospital" must be addressed to The Manager, "The Hospital," The Hospital Building, 28 and 29 Southampton Street, London, W.C.**

**Advertisements:**

To ensure insertion, all advertisements for The Hospital must reach the Manager not later than Wednesday morning in each week. For scale of charges see pages v and 4.

**Subscriptions** may begin at any time, and are payable in advance. Cheques and Post-Office Orders should be crossed London County and Westminster Bank, Covent Garden Branch, and made payable to the Manager of The Hospital.

Rates of Subscription (including postage).

| *United Kingdom.* | | *Foreign and Colonial.* | |
|---|---|---|---|
| Three Months ... ... | 2s. 0d. | Three Months ... ... | 2s. 6d. |
| Six Months ... ... | 4s. 0d | Six Months ... ... | 5s. 0d. |
| Twelve Months ... ... | 6s. 6d. | Twelve Months ... ... | 8s. 8d. |

**Remittances:**

**It is especially requested that remittances be made by Cheque or Postal Order, and in halfpenny stamps only when the amount is under sixpence.**

